# Considering the Closure of Arteriovenous Fistulas in Kidney Transplant Recipients

**DOI:** 10.34067/KID.0000000000000235

**Published:** 2023-08-31

**Authors:** Zhuotao Xiao, Joris I. Rotmans

**Affiliations:** Department of Internal Medicine, Leiden University Medical Center, Leiden, The Netherlands

**Keywords:** arteriovenous fistula, heart failure, hemodialysis access, kidney transplantation

Kidney transplantation (KTx) is considered the best treatment modality for most patients with ESKD because quality of life and survival are superior when compared with dialysis. However, because of the shortage of available kidney allografts, a large proportion of patients are being treated with hemodialysis (HD) until transplantation. A permanent arteriovenous (AV) access is the preferred type of vascular access for HD patients because it results in a lower complication rate when compared with central venous catheters. However, a functioning AV access might have a substantial effect on the transplant recipient while the access is no longer used for HD. In current clinical practice, the AV access is ligated in the vast minority of transplant recipients, as illustrated by Hick and coworkers, who reported about a cohort of >167,000 patients, in which only 4.6% of patients underwent AV access ligation after KTx.^1^ For most patients, the AV access is maintained after successful KTx in view of the potential risk of losing allograft function and the future need to restart HD.

In recent years, the management of AV access after KTx has become an emerging topic in the clinical and scientific field of HD access. In patients with clear symptoms related to their AV access, such as high-output heart failure, steal syndrome, or aneurysmal degeneration, clinical decision making is relatively easy, and the AV access is usually ligated. However, for asymptomatic patients, it is far more difficult to balance the pros and cons of maintaining a functional AV access. Indeed, various studies have pointed to the potentially detrimental effects of the AV access. These side effects mainly relate to its effect on the cardiovascular system, which already starts immediately after the creation of the AV access. Indeed, the reduced peripheral vascular resistance instantaneously leads to a rise in cardiac output. Because of this continuous hyperdynamic circulation, both left ventricular mass and pulmonary artery pressure increase. In some patients, the effective cardiac output decreases, leading to insufficient systemic perfusion,^2^ particularly in patients with high-flow fistulas. Impaired brain perfusion is one of the important manifestations that might occur, which can contribute to cognitive impairment.^3^ Hence, the management of AV access after KTx is an issue of great importance.

There is growing evidence suggesting that ligation of AV access can improve long-term cardiac function. The latest meta-analysis, including ten observational cohort studies, reported that AV access ligation could significantly decrease left ventricular mass index in KTx recipients.^4^ Meanwhile, this meta-analysis also reported that kidney graft function could benefit from AV access ligation. In 2019, Rao and coworkers published the results of the first randomized clinical trial in which KTx recipients with patent AV fistulas (AVFs) were randomized to AVF ligation or no intervention.^5^ All patients were transplanted >12 months ago, had a stable graft function, and had an average AVF flow of approximately 1500 ml/min. After ligation, the left ventricular (LV) mass and left atrial volume were reduced by 15% and 17.5 ml, respectively, and the serum N-terminal-pro-brain natriuretic peptide also decreased from 411 ng/L to 166 ng/L. Subsequently, the 5-year follow-up data confirmed the long-term benefit of AVF ligation on the LV mass index.^6^ Importantly, this study was not powered to study the effect of AV access ligation on physical performance or cardiovascular events.

With the improvement of kidney allograft prognosis, KTx recipients might have substantial cardiovascular benefit from AV access ligation. On the other hand, the advantages of AV access preservation are clear because it remains challenging to accurately predict allograft survival. Therefore, the question arises in which patients AV access ligation should be advocated. There is a large variation in clinical practice and no consensus among clinicians on this topic, as illustrated by a survey comprising eight case vignettes of asymptomatic KTx patients with a functioning AVF.^7^ In four of eight cases, <70% of the 585 respondents agreed on the management strategy for the AV access.

The study by Masson and coworkers^8^ adds important observations to this debate. In this study, 43 KTx recipients with a functional AVF were recruited and followed up to 48 months after AVF ligation. The AVFs of the patients were mostly located in the forearm (88.4%), and AVF closure was performed after a median time of 560 days after KTx. The median AVF flow was 750 ml/min. The investigators collected 24-hour ambulatory BP, serum cardiac biomarkers, serum creatinine, and transthoracic echocardiogram results for each patient. The investigators observed a significant decrease in LV end-diastolic volume, LV end-systolic volume, LV mass, interventricular septum diameter, and left atrial volume, in the first 6 months after AVF ligation, whereupon these cardiac morphometric parameters remained stable. N-terminal-pro-brain natriuretic peptide significantly dropped as well in the first 6 months after AVF ligation (345–230 pg/ml, *P* = 0.0001), suggesting an improvement in cardiac function. No significant change in LV ejection fraction, systolic BP, or eGFR was observed after AVF ligation while diastolic BP increased by 4.4 mm Hg.

This prospective study was well-designed and provided additional data relevant for the VA management after KTx. The observed effect on cardiac parameters was in line with the study of Rao,^5^ except for the effect on diastolic BP. It is noteworthy that the median flow volume of the fistula was 750 ml/min in the study by Masson, which is significantly lower than the median AVF flow in the study by Rao. This suggests that AVF ligation could also be meaningful in KTx patients with a relatively low AVF flow volume.

Some limitations of this study were also mentioned by the authors. Unfortunately, this study lacked a control group, which makes the results less convincing because of the potential interference of confounding factors. This effect might be small because the results of the study by Rao and coworkers revealed no change in the clinical, biological, and radiological parameters in their group without AVF ligation, during a follow-up of 6 months.^5^

Although this study revealed important observations, it does not put an end to the debate on AV access management after KTx. Thus far, the effect of AV access ligation on cardiovascular events or functional performance has not been studied. Such study would require a much larger number of participants because of the variability in patient prognosis and the multifactorial origin of cardiovascular diseases in KTx recipients.

What do we need to do in the meantime with a functional AV access in KTx recipients? We suggest making the AV access a parameter that should be monitored routinely after KTx. By doing so, patients with high-flow fistulas can be identified who are particularly at risk to develop (symptomatic) cardiac dysfunction. Whether to ligate a fistula in individual patients depends on access-related symptoms, their cardiac status, future options for vascular access, kidney allograft prognosis, and obviously patient preference, also regarding the preferred treatment modality in case of kidney allograft failure.
